# Multiple oncogenic viruses identified in Ocular surface squamous neoplasia in HIV-1 patients

**DOI:** 10.1186/1750-9378-5-6

**Published:** 2010-03-26

**Authors:** Kenneth O Simbiri, Masanao Murakami, Michael Feldman, Andrew P Steenhoff, Oathokwa Nkomazana, Gregory Bisson, Erle S Robertson

**Affiliations:** 1 Department of Microbiology, and Abramson Comprehensive Cancer Center, Tumor Virology Program, University of Pennsylvania, 202A Johnson Pavilion, 3610 Hamilton Walk, Philadelphia, PA 19104-6076, USA; 2 Pathology and Laboratory Medicine, Founders Building, 6.058 HUP/4283, 6th Floor, Philadelphia, PA 19104-6076, USA; 3 Botswana-University of Pennsylvania Partnership, The Children's Hospital of Philadelphia and the Center for AIDS Research, University of Pennsylvania, 3615 Civic Center Blvd, Suite 1202 ARC, Philadelphia, PA 19104-4318, USA; 4 University of Botswana School of Medicine PBag 0022, Gaborone; 5 Center for Clinical Epidemiology and Biostatistics, 832 Blockley Hall, 423 Guardian Dr. Philadelphia, PA 19104-6076, USA; 6 Department of Microbiology, Kochi Medical School, Kohasu, Nankoku, Kochi, 783-8505, Japan

## Abstract

**Background:**

Ocular surface squamous neoplasia (OSSN) is a rare cancer that has increased in incidence with the HIV pandemic in Africa. The underlying cause of this cancer in HIV-infected patients from Botswana is not well defined.

**Results:**

Tissues were obtained from 28 OSSN and 8 pterygia patients. The tissues analyzed from OSSN patients were 83% positive for EBV, 75% were HPV positive, 70% were KSHV positive, 75% were HSV-1/2 positive, and 61% were CMV positive by PCR. Tissues from pterygium patients were 88% positive for EBV, 75% were HPV positive, 50% were KSHV positive, and 60% were CMV positive. None of the patients were JC or BK positive. *In situ *hybridization and immunohistochemistry analyses further identified HPV, EBV, and KSHV in a subset of the tissue samples.

**Conclusion:**

We identified the known oncogenic viruses HPV, KSHV, and EBV in OSSN and pterygia tissues. The presence of these tumor viruses in OSSN suggests that they may contribute to the development of this malignancy in the HIV population. Further studies are necessary to characterize the molecular mechanisms associated with viral antigens and their potential role in the development of OSSN.

## Background

Ocular surface squamous neoplasia (OSSN) is a conjunctival or corneal neoplastic growth that encompasses the conditions of simple dysplasia to conjunctival intraepithelial neoplasia (CIN) to invasive squamous cell carcinoma [1]. Similar to cancer of the cervix, it has a relatively high rate of recurrence after treatment and may metastasize [2]. OSSN has gained interest in the past few years in its association with the HIV pandemic and its increase in incidence is collinear with the increase in HIV [3]. Prior to the HIV pandemic, OSSN was noted to occur predominantly in the elderly for whom it is the third most common oculoorbital tumor after melanoma and lymphoma [4,5]. In addition to advanced age and male sex, other risk factors linked to its pathogenesis have included ultraviolet light B rays and mutation of the p53 tumor suppressor gene [6], immunosuppression in organ transplant recipients [7], cigarette smoking, and in some settings, HPV infection [3,8]. In Africa, OSSN is becoming more common, more aggressive, and more likely to affect young people, especially females [6].

In parallel with the dramatic increase of HIV in Africa, several countries have noted a sharp rise in the incidence of OSSN in HIV infected individuals such that OSSN is currently the most common ocular tumor among adults in Africa [6,9]. Africa has the highest rate of HPV infection in the world, with an age-adjusted prevalence of 25.6% in women aged 15-74 years, followed by South America (14.3%), Asia (8.7%), and Europe (5.2%) [10]. In 2006, Waddell et al. investigated the role of HIV infection in the etiology of corneo-conjunctival intraepithelial neoplasia (CIN) and carcinoma [11]. The prevalence of HIV infection in these cases was 64% compared to 31% in controls [11]. These findings demonstrated a strong association between increased incidence of OSSN and HIV-1 infection as applied to all tumor stages in Uganda. HIV-positive participants were markedly immunosuppressed at the time of diagnosis and their early mortality was high [11]. The majority of the tumors were in the interpalpebral area of the conjunctiva, which supports the concept of UV radiation as a co-factor in the etiology of OSSN [11].

Pterygium is a raised benign growth on the eye that is most often directly related to over-exposure to the sun and dry, dusty conditions may also be a factor [12]. Pterygium in the conjunctiva is characterized by elastotic degeneration of collagen and fibrovascular proliferation, and in some cases by "wing-like" conjunctival overgrowth of the cornea [13]. Some studies have investigated human papillomavirus (HPV) as a risk factor for the development of pterygia [14,15]. Interestingly, recent studies have shown that pterygia can develop into OSSN, and thus may be considered a pre-malignant form of OSSN [16].

The aim of this study was to identify the viral etiologic agent(s) associated with OSSN in the HIV-infected population in Botswana. There are numerous examples of associated malignancies in the HIV population, which include CNS lymphomas with greater than 95% Epstein Barr Virus (EBV) positivity [17], pleural effusion lymphomas which are usually co-infected with both tumor gammaherpesviruses EBV and Kaposi's sarcoma associated herpes virus (KSHV) [18,19], AIDS associated B and T cell lymphomas [20], Kaposi's sarcoma (KS)[21], Burkitt's lymphoma (BL) [22], anogenital carcinomas and papillomas [23], oral cancers [23,24] osteosarcomas [25] mesotheliomas [25] and brain cancers [2,18,21,23,26-28]. Also, the oncogenic papova virus Human papillomavirus (HPV) infects keratinocytes causing benign and malignant tumors [1]. HPV proteins E6 and E7 are oncogenic and have been associated with cell immortalization and antiapoptotic effects. The HPV types 6, 11, 16, 18 and 33 have been identified in benign and malignant conjunctival lesions [3].

The role of chronic latent viruses and their intermolecular interactions in combination with other environmental factors in the context of HIV infection is likely to be important for initiating and maintaining HIV-associated malignancies. However, a causal role for these viruses is difficult to ascertain at this time. Nevertheless, infection with a range of viruses from the herspesviridae family including HSV, HCMV, EBV, and KSHV may contribute to the oncogenic process in OSSN, as has been observed with other human cancers [29].

## Methods

### Patients

HIV-1 infected patients with conjunctival lesions seen at Princess Marina Hospital, Gaborone, Botswana from April 11 2007 to April 14 2008 were enrolled in this study (IRB #805049 and Ministry of Health, Botswana REF NO: PPME 13/18/1 Vol III 141). Briefly, patients were identified by an ophthalmologist in the tertiary care ophthalmology clinic at Princess Marina Hospital which serves as the referral center for southern Botswana. All HIV-1 positive patients diagnosed using a HIV-ELISA (Abbott Laboratories, Hoofddorp, the Netherlands) with clinical features suggestive of OSSN or pterygium were enrolled the day before surgery. Written consent was obtained from each subject in either Setswana or English (according to subject's preference). Tissue specimens obtained in the ophthalmology operating room were divided into two pieces by the ophthalmology surgeon - one piece was sent for histopathologic analysis and the other was immediately placed in tissue transport medium for shipment to the University of Pennsylvania viral oncology laboratory. Histologic confirmation of each clinical diagnosis was obtained from the Botswana National Health Laboratory's histo- pathologist. 39 patients participated in the study, however tissue samples of varying sizes were obtained from only 36 cases. Tissues from patients included in this study were 28 whose clinical diagnosis was OSSN and 8 with pterygia (Table 1). Only patients who signed the University of Pennsylvania and Botswana Ministry of Health Institutional Review Board consent forms were included in this study. In this study all patients were enrolled from the Botswana HIV clinic. Therefore we were not able to obtain conjunctiva controls from HIV-1 negative patients or those diagnosed with OSSN and pterygia identified as HIV negative.

**Table 1:** Patient characteristics

Case #	Diagnosis	Sex	Age	CD4 count	HIV Viral load	Affected Eye	ARV Status	Histology Results
1	OSSN	M	45	40	<400	Right	+	SCC

2	Pterygium	M	49	373	<400	Left	+	Pterygium

3	OSSN	M	39	64	<400	Right	+	Pterygium

4	OSSN	M	32	521	ND	Right		SCC

5	OSSN	F	47	44	ND	Right	+	SCC

6	OSSN	M	43	174	120,000	Right		SCC

*7	Pterygium	F	29	ND	ND	Right	+	Pterygium

8	OSSN	F	42	134	120,000	Left	+	SCC

9	OSSN	F	27	171	<400	Left	+	NA

*10	OSSN	M	40	ND	<400	Right		Severe dysplasia

11	OSSN	M	38	326	<400	Left	+	Severe dysplasia

12	OSSN	F	22	457	ND	-		NA

13	OSSN	F	44	ND	<400	Left	+	SCC

14	OSSN	F	39	200	<400	Left	+	Pterygium

15	Pterygium	F	38	725	ND	Left		Pterygium

16	Pterygium	F	40	600	ND	Left		Pterygium

17	Pterygium	F	38	491	ND	Left		Pterygium

18	OSSN	F	44	314	ND	Left		NA

19	OSSN	F	37	56	<400	Right	+	Pterygium

*20	OSSN	M	48	21	<400	Right	+	SSCC

21	OSSN	F	49	ND	ND	Right		NA

22	OSSN	F	30	90	ND	Right		SSCC

23	OSSN	F	36	220	<400	Right	+	Severe dysplasia

24	OSSN	F	35	ND	644,000	Right	+	Mild dysplasia

25	Pterygium	F	39	293	<400	Left	+	Pterygium

26	Pterygium	M	47	546	<400	Right		Pterygium

27	Pterygium	F	31	113	ND	Left	+	Pterygium

28	OSSN	M	50	87	<400	Right	+	SCC

29	OSSN	F	34	192	ND	Left		SCC

30	Pterygium	F	29	70	<400	Right	+	Pterygium

31	OSSN	M	45	62	17,000	Left	+	NA

32	OSSN	F	28	107	<400	Left	+	Severe dysplasia

33	OSSN	F	42	236	342	Left		Pterygium

34	OSSN	M	33	94	81,972	Left	+	SCC

35	OSSN	M	40	64	685,000	Right	+	Cancer in situ

36	OSSN	F	36	38	200,000	Right	+	NA

37	OSSN	F	49	ND	ND	Right		Severe dysplasia

38	OSSN	F	24	31	ND	Left	+	Moderate dysplasia

39	OSSN	F	44	121	ND	Right		SCC

* Tissue not available, NA - Not available

The table shows patient diagnosis, sex, age on entering study, CD4 counts, viral load, the eye affected, the retroviral therapy status of each patient, and histology results. Tissues were not obtained from 3 patients and indicated with an asterisk. Patients #s 3, 14, 19, and 33 were diagnosed as OSSN but histology results indicated that they were pterygia.

### DNA extraction, *in situ *hybridization and immunohistochemistry of tissue samples

Total cellular DNA was extracted from fresh OSSN and pterygia tissues by digestion with proteinase K and phenol-chloroform extraction as previously described [30].

**Figure 1:**
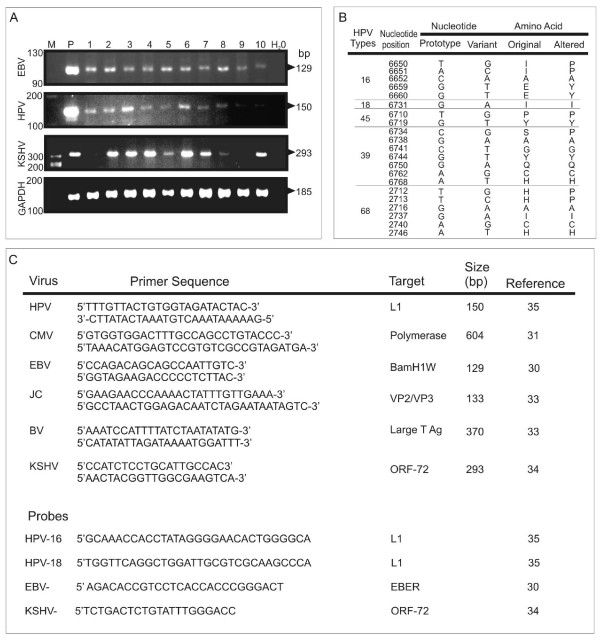
**PCR analysis of OSSN for detection of human viruses**. **A**. Representative PCR analysis of tumor virus detection from OSSN tumor samples. For EBV PCR, LCL-2 10^5 ^cells which contain 20-50 copies of EBV per genome were used as positive control and distilled water as control for PCR mix. For HPV, Hela 10^5 ^cells which contain 10-20 copies of HPV-18 per genome were used as positive control and distilled water as control for PCR mix. Similarly for KSHV we used BCBL1 cells which contain 30-50 copies of KSHV per genome as positive control and distilled water as control for PCR mix. The PCR products are shown after electrophoresis on a 3% agarose gel containing 100 ng/ml ethidium bromide. PCR product size for each virus type is shown on the right side. We show representative gels showing the PCR results of which EBV was detected in 83% of the samples, HPV in 75% of the samples, KSHV in 70% of the samples and CMV in 61% of the samples. We did not detect JC and BK virus in any sample. **B**. Representative point mutations in HPV-L1 nucleotide and amino acid sequence variation in OSSN biopsies. We detected HPV-16, 18, 45, 39, and 68 isolates. As shown, the changes were mainly missense and nonsense mutations. **C**. Shows the primer sequences used in the PCR analysis of the oncogenic viruses in OSSN and pterygia tissues and probes used for *in situ *hybridization.

PCR analysis was performed as previously described [31-35] with each virus specific primer (see Figure 1C).

To support our PCR findings for the presence of HPV, EBV, and KSHV in tissues we performed *in situ *hybridization and immunohistochemistry on 5 μm thick paraffin-embedded sections to detect virus specific antigens. We noted alignment with the probes for HPV-16-L1 at 481-511 bp (accession DQ155283), EBV-EBER at 1-26 bp (accession J02078) and for KSHV-ORF-72 at 201-220 bp (accession 009333). *In situ *hybridization probes for HPV-16 or 18 [36], EBV-EBER [30], and KSHV-ORF-72 [34] were labeled with digoxigenin-ddUTP (Dig) using a commercial kit (Roche Inc., Indianapolis, IN) and used as described previously [37]. We used commercial antibodies HPV 16-L1 and HPV 18-E6 (DAKO Inc., Carpentaria, CA), and monoclonal antibody S12 for EBV-LMP1, and monoclonal antibody derived from KSHV encoded LANA for immunohistochemistry [38]. Results from the ISH and IHC were analyzed by a licensed histopathologist at the University of Pennsylvania, School of Medicine.

### DNA Sequencing

To determine the HPV types and variants, analysis of the PCR products were directly sequenced on an automated sequencer according to manufacturer's protocol (Applied Biosystems Inc., Foster City, CA). Briefly, HPV PCR products with consensus primers were prepared and the reaction was run under standard cycle conditions of 96°C for 10 seconds, 55°C for 5 seconds, and 60°C for 4 minutes for a total of 25 cycles. The PCR amplicons were then ethanol precipitated, dried briefly in a speed-vacuum, resuspended in Hi Di formamide and loaded onto the 3730 DNA analyzer (Applied Biosytems Inc., Foster City, CA) for capillary electrophoresis and analysis. Sequence analysis was done using Applied Biosystems Sequence Analysis Software v5.3.1. The sequences were compared to the specific HPV prototype sequences in GenBank.

## Results

### Patients were HIV positive and predominantly female

**Figure 2:**
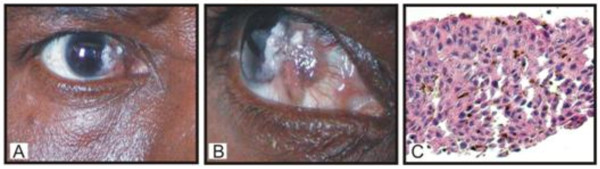
**A. Representative photograph showing OSSN (A) and a closer view (B), C**. H&E staining showing features of *in situ *carcinoma characterized by full thickness changes in nuclear: cytoplasm ratio, nuclear pleomorphism, dyskeratotic cells, and presence of koilocytes.

Tissue samples, sera, and plasma were collected from 39 patients with a diagnosis of OSSN or pterygium from Princess Marina Hospital in Botswana (Table 1). All patients were HIV-1 positive and 24 were on antiretroviral therapy (ARV) at the time of the study. Of the 39 cases, 13 were male and 26 were female, 30 had OSSN, and 9 had pterygia. The patients were young with 38 cases under the age of 50 years old. CD4 counts were available for 32 patients and 20 patients had counts below 200 cells/ml when ARVs were started. Only 36 tissue samples were received for processing-28 OSSN and 8 pterygia. Histological analysis was obtained on the samples except for six where no histological information was provided, and indicated that some of the tumors were squamous cell carcinoma (SCC), surface squamous cell carcinoma (SSCC), pterygia, severe dysplasia, moderate, and mild dysplasia (Table 1). No tissues were obtained from cases # 7, 10, and 20 and so no further analyses were done. The patients on ARVs had viral loads that were generally low. However, a few had high copies (> 100,000 copies/ml) and were patients started on ARVs therapy relatively recently. Viral load analysis was not completed for fifteen patients. No cases were bilateral, however there was a preponderance for the left eye for the few pterygium cases. The photographs represent example showing a patient with OSSN (2A), and a closer view (2B). H and E staining of OSSN tissues typically shows *in situ *carcinoma characterized by full thickness changes in nuclear to cytoplasm ratio, nuclear pleomorphism, dyskeratotic cells, and the presence of koilocytes from HPV infection that could be seen in some lesions (Figure 2C).

### PCR analysis detected DNA from oncogenic viruses in OSSN tissues

We performed PCR analysis on all tissues obtained to determine whether any tumor viruses are associated with OSSN. As shown in table 2, 30 (83%) cases were EBV positive, 27 (75%) cases were HPV positive, 25 (70%) cases were KSHV positive, 22 (61%) cases were CMV positive, 25 (70%) were HSV 1/2 positive, and none of the patients had BK or JC. Using specific primers for identification of specific HPV types, we detected HPV- 6, 11, 16, 18, 31, and 33 (Table 3). The table shows that in both OSSN and pterygia samples HPV-11, 16, and 18 were the most common. HPV-6, 31, and 33 were rare and not detected in pterygia samples. Results from the PCR analyses are depicted by the representative sample of cases tested for EBV, HPV and KSHV (Figure 1A). It is to be noted that a majority of the cases tested were positive for all three oncogenic viruses, albeit with different levels of intensity based on difference in viral loads, cancer stage, and tissue size from which the DNA was extracted. Some of the tissues were co-infected with HPV/EBV/KSHV in different combinations as determined by PCR (Table 2, and Figure 1). Interestingly, the data showed that the majority of the samples had signals for both HPV-16 and 18 with only 21% of OSSN positive for HPV-6 and 46% with signals for HPV-11 (Table 3). Moreover, 25% of OSSN tissues were positive for HPV-31 and 4% of OSSN tissues were positive for HPV-33 (Table 3). Therefore, in the OSSN tissues from immunocompromised patients there was a high degree of co-infection with multiple oncogenic viruses as indicated by PCR, IHC, and ISH (Table 4). Importantly, it should also be noted that all PCR analyses done on archival tissues were negative or inconclusive and therefore were not included in this study (data not shown). This was likely due to the inadequate or compromised conditions of the stored tissues.

**Table 2:** Oncogenic viruses identified by PCR in patient samples

Case #	Diagnosis	HPV	EBV	KSHV	CMV	BK	JC	HSV 1/2
1	OSSN	+	+	-	-	-	-	+

2	Pterygium	+	+	+	+	-	-	+

3	OSSN	+	+	+	+	-	-	+

4	OSSN	+	+	+	-	-	-	+

5	OSSN	+	+	-	+	-	-	+

6	OSSN	+	+	+	+	-	-	+

8	OSSN	+	+	+	-	-	-	+

9	OSSN	+	+	-	-	-	-	+

11	OSSN	+	+	+	-	-	-	-

12	OSSN	+	+	+	-	-	-	-

13	OSSN	-/+	+	-	+	-	-	-

14	OSSN	-	-	+	+	-	-	+

15	Pterygium	-	+	+	+	-	-	+

16	Pterygium	-	+	-	+	-	-	+

17	Pterygium	-	+	+	-	-	-	+

18	OSSN	-	+	+	+	-	-	+

19	OSSN	-	+	+	-	-	-	-

21	OSSN	-	+	+	-	-	-	-

22	OSSN	-	+	-	-	-	-	-

23	OSSN	-	+	+	+	-	-	+

24	OSSN	-	+	+	+	-	-	-

25	Pterygium	-	+	-	+	-	-	+

26	Pterygium	+	-	+	+	-	-	-

27	Pterygium	+	+	+	-	-	-	+

28	OSSN	+	+	-	-	-	-	+

29	OSSN	+	-	+	+	-	-	+

30	Pterygium	+	+	-	+	-	-	-

31	OSSN	+	+	+	+	-	-	+

32	OSSN	-	+	-	+	-	-	+

33	OSSN	+	-	+	+	-	-	+

34	OSSN	+	+	+	-	-	-	-

35	OSSN	+	-	+	-	-	-	-

36	OSSN	+	+	-	+	-	-	+

37	OSSN	+	-	+	+	-	-	+

38	OSSN	+	+	+	+	-	-	+

39	OSSN	+	+	+	+	-	-	+

Oncogenic viruses identified in each OSSN and pterygium patient as determined by PCR analysis. Most cases tested positive for HPV, EBV, KSHV, and HSV. HPV results for samples 14-24 were intriguing since most were positive on IHC and ISH. None was positive for BK and JC.

**Table 3:** Proportion of HPV types identified by PCR in OSSN and Pterygium tissues

Virus Type	OSSN	Pterygium
HPV-6	21%	0

HPV-11	46%	63%

HPV-16	61%	75%

HPV-18	54%	63%

HPV-31	25%	0

HPV-33	4%	0

Proportion of HPV types identified in patient samples by use of type specific HPV primers, HPV types 6, 11, 16, 18, 31, and 33. It is noted that HPV-11, 16, and 18 were common in both OSSN and pterygia cases, with HPV-6, 31, and 33 only detected in OSSN cases.

**Table 4:** Oncogenic viruses identified in each patient by IHC and ISH

Case #	Diagnosis	HPV			EBV			KSHV		
		
		IHC	ISH	PCR	IHC	ISH	PCR	IHC	ISH	PCR
1	OSSN	-	-	+	+	+	+	+	+	-

2	Pterygium	+	+	+	+	+	+	+	+	+

3	OSSN	+	+	+	+	-	+	+	-	+

4	OSSN	+	-	+	+	+	+	+	+	+

5	OSSN	+	+	+	-	+	+	+	+	-

6	OSSN	+	+	+	+	+	+	+	+	+

8	OSSN	+	+	+	+/-	+/**-**	+	**-**	**-**	+

9	OSSN	+	+	+	+	+	+	+	+	-

11	OSSN	+	+	+	+	+	+	+	+	+

12	OSSN	+	+	+	+	+	+	+	+	+

13	OSSN	-/+	-/+	-/+	+	-	+	+	+	-

14	OSSN	+	+	-	+	+	-	+	-	+

15	Pterygium	+	+	-	+	+	+	+	+	+

16	Pterygium	+	+	-	+	-	+	+	+	-

17	Pterygium	+	-	-	+	+	+	+	+/-	+

18	OSSN	+	+	-	+	+	+	+	+	+

19	OSSN	+	+	-	+	+	+	+	+	+

21	OSSN	+	+	-	+	+	+	+	+	+

22	OSSN	+	+	-	+	+	+	+	+	-

23	OSSN	+	-	-	+	+	+	+	+/-	+

24	OSSN	+	+	-	+	+	+	+	+	+

25	Pterygium	+	+/-	-	+	+	+	+	+	-

26	Pterygium	+	+	+	+	+	-	+	-	+

27	Pterygium	+	+	+	+	+	+	+	+	+

28	OSSN	+	+/-	+	+	+	+	+	+	-

29	OSSN	+	+	+	+	+	-	+	+	+

30	Pterygium	+	+	+	+	+	+	+	+	-

31	OSSN	+	+	+	-	-	+	+/-	+/-	+

32	OSSN	-	-	-	+	+	+	+	+	-

33	OSSN	+	-	+	+	+	-	+	-	+

34	OSSN	-	+	+	+	+	+	+	+	+

35	OSSN	+	+	+	+	+	-	+	+	+

36	OSSN	+	+	+	+	-	+	+	+	-

37	OSSN	-	-	+	+	+	-	+	+	+

38	OSSN	+	+	+	+	+	+	+	+	+

39	OSSN	+	+	+	-	-	+	+	+	+

+/- Indeterminate

Oncogenic viruses identified in each patient by Immunohistochemistry and *In situ *hybridization. It is noted that for each of the samples analyzed for the specific viruses-HPV-16/18, EBV, and KSHV, there was high concordance in IHC and ISH. For each virus a column for PCR results has been added for comparison. Due to lack of specimen we were unable to test for the other viruses. No tissue samples were available for patients 7, 10, and 20.

### *In situ *hybridization analysis detected signals from multiple oncogenic viruses

**Figure 3:**
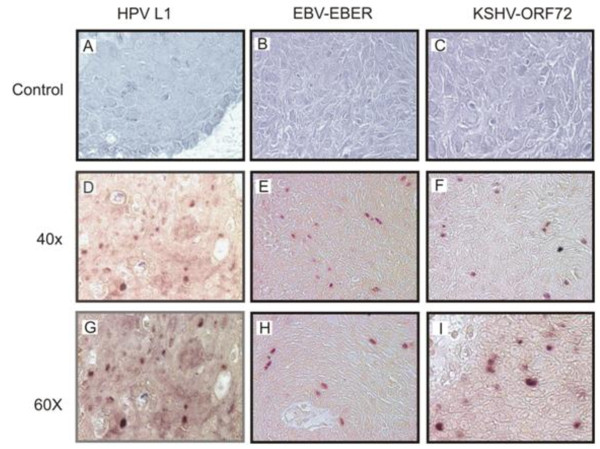
***In situ *hybridization**. Panels **A-C **shows results from Sense probe negative controls with no detectable nuclear staining. Panels **D-F **the positive cells of tissue sections probed with HPV-L1, EBV-EBER, and KSHV-ORF72 showed dark brown staining of the nucleus. The probes did not detect all viruses in all tumor cells, however, HPV, EBV, and KSHV were detected in some regions. Panels **G-I **shows a higher magnification of 60× compared to panels **D-F **which showed magnification of 40×.

*In situ *hybridization was performed on sections from fresh paraffin-embedded tissue utilizing HPV-16 L1, EBV EBER, and KSHV ORF-72 specific oligonucleotide probes to corroborate the above results, the specific probes were detected using digoxigenin dd UTP label. However, we did not perform ISH for CMV, HSV 1/2, and other types of HPV due to insufficient tissue sections. We observed punctate and diffuse nuclear signals indicative of integrated and episomal viral DNA genomes respectively, as seen in the representative Case #31 photographs (Figure 3D-F and 3G-I). The punctate and episomal viral nuclear signals were more obvious in HPV samples. Importantly, no signals were observed with non specific probes or sense probes used as controls (Figure 3A-C). These probes for EBER-sense, KSHV-sense and HPV non-specific were used to clearly demonstrate that these signals obtained were due to the specific signals from the viral encoded transcripts and was unlikely to be due to non-specific interactions from cellular transcripts or deposited label. It should be noted that the intensity of the *in situ *hybridization staining was somewhat variable with some of the positive cells closer to the surface of the lesions with clear nuclear or perinuclear staining within the epithelial but little or no detectable signals in the subepithelial layers and the remaining epithelial tissue. For additional tissues diagnosed as OSSN, similar analyses were done and showed specific signals for the HPV-L1, EBV-EBER, and KSHV-ORF-72 for some of the cases. The results suggest similar patterns of infection with these viruses and correlated nicely with the PCR data from the samples obtained (Table 4).

### Immunohistochemical Analysis showed positive signals for viral antigens in OSSN tissues

**Figure 4:**
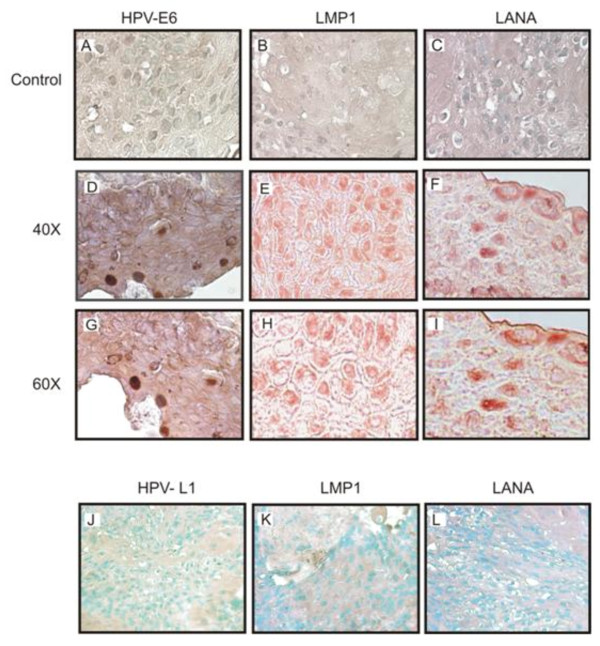
**Representative Immunohistochemistry results of selected OSSN tissues**. Panels **A-C **shows no nuclear staining in the negative controls, Panels **D-F **show positive cells as dark brown staining of the nucleus with HPV-E6 antibody, as well as positive staining for LMP1 and LANA using EBV-LMP1 hybridoma supernatant and KSHV-LANA specific antibody. Panels **G-I **shows a higher magnification of 60× compared to panels D-F taken at 40×. Panels **J-L **are immunohistochemistry results for negative samples showing no detectable staining which would represent positive signals.

Based on PCR and *in situ *hybridization results in which we detected viral genes in the DNA extract and tissues of OSSN, we performed immunohistochemistry using specific antibodies to identify viral antigens known to be expressed by these viral agents in tissues. We noted specific signals with the antibodies to HPV- E6, EBV-LMP1, and KSHV-LANA (Figure 4, panels D-I) from representative Case # 31 compared to the controls. The cells showed strong dark staining of the nuclei suggesting positive signals for the viral antigen E6, and staining for LMP1, and LANA respectively. The cells that were intact showed dramatic signals suggesting the presence of multiple viruses in this cancer. Positive immunostaining was largely confined to the nuclei of the infected superficial epithelial layers of the tissues for E6 and LANA but also showed signals throughout the cell membrane or in focused regions of the membrane as would be expected for LMP1. To determine the specificity of the signals immunohistochemistry controls were performed without primary antibody (Figure 4, Panels A-C) and using tissue sections demonstrated to be negative for EBV, KSHV, and HPV by PCR (Figure 4, Panels J-L). The results showed no detectable signals for any of the sections developed (Figure 4, panels A-C, and J-L respectively) and supports the presence of these 3 oncogenic viruses in the tissues tested that were diagnosed as OSSN in HIV patients.

### Multiple types of HPV were identified in individual patients

**Figure 5:**
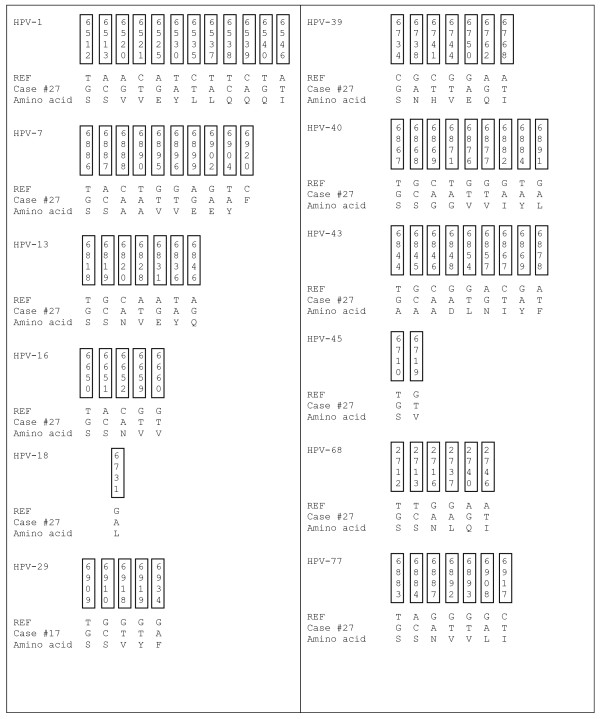
**Nucleotide and amino acid sequence variation in OSSN and pterygia patients**. Shown are representative samples of the most common HPV types detected by sequencing using GP5+/GP6+ primers of the L1 region. The table shows nucleotide variation and amino acid changes. The top 4 rows show the nucleotide position, the 5^th ^row shows the HPV reference nucleotide sequence, the 6^th ^row shows the variation in patient nucleotide sequence, and the 7^th ^row is the resulting variant amino acid sequence. In each HPV type, the variations were the same for each study case in the selected nucleotide sequence with some differences upstream and downstream in the different subjects, except case #15 which varied in a specific nucleotide in all HPV types, besides HPV-1.

Amplicons from PCR products were further purified and sequenced using the consensus primers GP5+/GP6+. We analyzed a 150-bp fragment of the L1 region of 36 cases for specific HPV types and variations. Using a 40 base sequence including the GP5+ priming site selected as the signature for each HPV type online BLAST sequence alignment algorithms of the L1 3' end. The results suggest that based on these analyses twenty six HPV types were identified, with HPV- 7, 13, 16, 18, 39, 43, and 45 being the most common (Table 5). Importantly, all the tissues were positive for multiple HPV types based on the analyses. We identified nucleotide variations in the majority of samples. The variations were mainly missense and silent mutations whose significance in maintenance of the virus is yet to be determined (Figure 1B). However, we observed that for HPV 39, the L1 C6741T variation was similar to C6733T and C6903T [39] in cervical cancer in South American population, and for HPV 18, the L1 G6731A variation was similar in cervical cases in Africa at G6579A, G6749A, and G6917A, while for HPV16, the L1 T6650G and A6651C similarity was noted in cervical cancer cases from India at T6889G and A6667C [40], (Figure 5).

**Table 5:** HPV Types detected in patient samples by sequencing using consensus primers GP5+/GP6+

Case #	HPV type
1	7,13,16,18,39,40,43,45,59,61,68,70,77,85,91,97

2	7,13,16,18,39,40,43,45,59,61,68,70,77,85,91,97

3	7,13,16,18,39,40,43,45,59,61,68,70,77,85,91,97

4	7,13,16,18,39,40,43,59,61,68,70,77,91,97

5	7,13,16,18,39,40,43,45,59,61,68,70,77,85,91,97

6	7,13,16,18,39,40,43,59,68,70,77,91,97

8	7,13,16,18,29,39,40,43,45,59,61,68,70,77,85,91,97

9	7,13,16,18,39,40,43,45,59,61,68,70,77,85,91,97

11	7,13,16,18,39,40,43,45,59,61,68,70,77,85,91,97

12	16,18,39,40,43,45,59,61,68,70,77,85,91,97

13	7,13,16,18,39,40,43,45,59,61,68,70,74,85,91,97

14	16,18,29,39,40,43,45,59,68,70,85,91,97

15	7,13,16,18,39,40,43,45,59,61,68,70,77,85,91,97

16	7,13,16,18,39,40,43,45,59,61,68,70,74,77,85,91,97

17	7,13,16,18,29,39,40,43,45,59,61,68,70,77,85,91,97

18	7,16,18,39,40,61,70,77,85,91,97

19	16,18,39,40,43,45,61,70,77,85,91,97

21	7,13,16,40,45,70,77,85

22	7,13,16,39,43,45,70,85

23	7,13,43,45,70,77,85

24	7,43,45,85

25	13,16,18,39,40,43,45,59,61,68,70,77,85,91,97

26	13,16,18,39,40,43,45,59,61,68,70,77,85,91,97

27	1,3,7,11,13,16,18,39,40,43,45,59,61,68,70,77,85,89,91,97

28	29,43,45,59,85

29	45,59,68,70,85

30	1,3,7,11,13,16,18,39,40,43,45,59,61,68,70,77,85,89,91,97

31	16,18,43,45,59,68,70

32	16,18,43,45,59,61,68,70

33	1,13,16,18,39,40,43,44,45,55,59,61,68

34	7,13,16,18,39,43,45,59,61,68,97

35	7,16,18,39,40,43,45,59,61,68,74

36	16,18,39,45,59,61,68,77,97

37	7,13,16,18,39,43,45,59,61,68,77,97

38	7,13,16,18,39,40,43,44,45,55,59,68,74,77,97

39	7,18,39,40,43,59,61,68,74,77,91,97

HPV types identified in patient samples by sequencing with the consensus primer GP5+/GP6+. The most commonly identified HPV type in both OSSN and pterygium tissues included the high risk HPV-18, 16, 45, 39, 59, and 68.

## Discussion

A dramatic increase in OSSN has been noted in sub-Saharan Africa over the last 10 years in HIV population [1]. In Uganda there has been an increase in incidence especially among young people linked to HIV infection [6]. In Botswana, data from Princess Marina Hospital in Gaborone currently indicates a pronounced increase in incidence of OSSN in young HIV-1 infected individuals. This initial study concurrently detected the presence of multiple tumor viruses in OSSN tissues. For the first time we showed the presence of HPV, EBV, KSHV, and CMV in ocular tissues in HIV/AIDS patients. Association of viruses with human cancers have already been reported, such as KSHV with KS and PEL [41], EBV with primary CNS lymphoma [17], nasopharyngeal carcinoma [26], and Burkitt's lymphoma [22], HPV with cervical cancer [2], anal cancer [42], and testicular cancer [43], However, the level of contribution by HIV to the development of human malignancies remains unclear and is likely to be through several mechanisms.

Infection and establishment of latency are mediated by viral proteins expressed in infected cells. Importantly, the immunomodulatory mechanisms used by one virus may actually enhance or benefit the activity or replication of other viruses. Many of these viruses (for example, HPV, CMV, and EBV) are quite common in the human population [44], but do not always lead to development of malignancies in healthy individuals with a competent immune system. However, infection with HIV resulting in immunosuppression may eventually tip the balance in favor of the tumor viruses, leading to an increased risk of cancer in the immune compromised individuals. The localization of these oncogenic proteins in the OSSN tissues show association, however establishment of a causal effect by any of the viruses, or a multiple of the viruses in an HIV atmosphere has yet to be fully confirmed. Determining the pathways involved in the initiation and establishment of the cancers in HIV patients compared to non-HIV patients will give us a better understanding of the interplay of the different oncogenic viral proteins within the normal immune system and immunosuppressed system. Though we have shown the presence of these viral proteins in the OSSN tissue, some investigators have not identified viral sequences in squamous cell conjunctival carcinoma (SCCC) which has also increased in the HIV infected population and has been suspected of having viral etiology [45,46]. Therefore, more studies are clearly needed to fully establish a role for these viruses in OSSN.

The localization of different tumor viruses in similar tissue sections suggest that these viruses may functionally interact to contribute to the OSSN phenotype. Similar to our findings of HPV and EBV in ocular tissues, Prayitno reported that in 19 cervical carcinoma samples 89% were positive for HPV and 68% were positive for EBV [47]. Nakamura et al suggested that HPV probably does not act alone in initiating ocular neoplasia, but that other factors including the ultraviolet light were involved [37]. Whereas studies involving HPV and cervical cancer have been associated with oncogenic HPV-16 and 18, our study identified HPV-6, 7,16, 18, 31, 45, and 97 in DNA from the OSSN and pterygia tissues as determined by PCR. Interestingly, we detected nucleotide sequences from HPV-1, 3, 7, 13, 16, 18, 29, 31, 33, 39, 40, 43, 44, 45, 55, 59, 61, 68, 70, 74, 77, 85, 89 91, and 97 by sequencing PCR amplicons from the different tissues. Some of the sequence alignments had scores greater than 80% with specific HPV types indicating presence of these HPV types or their variants in some of the samples. This suggests exposure to many types of HPV in this population, some of which are oncogenic, which with other factors may trigger cell proliferation leading to OSSN and other cancers in the immunocompromised patients. The selection pressure that may exist as a consequence of competition between opportunistic infections may further lead to mutations occurring in the HPV types as well as the other infectious agents. Interestingly, there was a significant amount of amino acid variation within the cases to suggest different HPV types when each sequence was aligned with the specific HPV prototype.

To corroborate the PCR results we performed *in situ *hybridization and immunohistochemistry studies. These results showed that in the invasive OSSN tissues the viruses were localized in similar tissue sections. Some of the viral genomes are likely to be integrated by their punctate signals and episomal viral DNA by their diffuse signal throughout the nucleus [24]. The number of cells positive for viral signals were modest, possibly a factor of the limited size of tissues tested, relatively small size of the tissue obtained for analysis, and lower sensitivity compared to PCR. Previous reports have suggested that infection with more than one HPV type enhances cervical cancer and oral squamous carcinoma [23]. Also, Mirzamani et al reported co-infections with EBV and HPV-16/18 in nasopharyngeal carcinoma (NPC) patients and concluded that both viruses were important in contributing to the pathogenesis of NPC [26]. It is not known if cases with OSSN without HIV infection had similar outcome with HPV and other viruses as we were unable to obtain these tissue samples. Moubayed et al reported HPV-6/11, HPV-16, and HPV-18 in a Tanzanian study, with the conclusion that it was HPV-16 and HPV-18 that was associated with OSSN [48]. They noted that the intensity of *in situ *hybridization was highly variable ranging from barely detectable to a few grains over some cells located near the surface of the lesions [48]. As in some of our hybridization results they noted nuclear or perinuclear staining within the epithelial lesions and a complete absence of hybridization signals in the subepithelial layers and the surrounding unaffected epithelium.

The significance of all the HPV types identified with respect to OSSN oncogenesis in individual patients, and specifically the infection with multiple oncogenic HPV types-both high risk and low risk, EBV, and KSHV viruses requires careful examination. Some studies have indicated that the oncogenic capacity of HPV variants may also differ between geographic regions based on HLA allele distribution in these populations [40]. The unknown impact of HLA allele differences, HPV variants, and other oncogenic viruses in this HIV-1 infected population is of significance and warrants further investigation.

In tropical countries secondary immunodeficiency attributable to chronic inflammation caused by parasitic or viral infections might contribute to tumorigenesis. The high frequency of coinfection of HPV, EBV, KSHV, CMV, and HIV in the OSSN patients may be a result of cooperative and complementary interactions of the viruses. In addition, we suggest that the disease might be more aggressive in HIV patients due to their immune dysfunction, abnormal cytokine and chemokine expression, growth factor production and exposure to ultraviolet rays. In this sample we cannot determine if the patients were initially infected with HPV, or HIV and other oncogenic viruses before occurrence of OSSN, or if some of the viruses were opportunistic infections as a result of OSSN disease or HIV infection. The effect of latency in ubiquitous viruses like EBV, HPV and high prevalence of sexually transmitted HSV and KSHV is not clear in this OSSN population. It has been reported, and we also note that the course of OSSN is more rapid in HIV infected patients and the occurrence is increasing in younger patients with HIV at a higher rate [3,6]. In HIV infected patients, it is not known at what stage OSSN occurs. Spitzer et al in a Malawi study reported that OSSN was the first detectable sign of HIV infection in the majority of cases [7].

## Conclusion

This study investigating the link between oncogenic viruses in OSSN in the HIV population suggests that multiple viral agents may have a role in development of this disease. The interaction between different viral antigens and their regulatory activities in immunocompromised individuals will contribute to cell survival and proliferation in the infected cells. Clearly, further study is warranted to understand the molecular processes involved in the increased incidence of OSSN in the HIV population.

## Funding

This work was supported by the UPENN Center for AIDS Research (P30-A1455008 to JH); the pilot project award to ESR and GB and the Botswana UPENN partnership in Gaborone, Botswana; Public Health awards from the National Cancer Institute of the National Institutes of Health (CA137894, CA138434, CA72150, CA91792, CA91792-S1, CA91792-S2, CA108461 to ESR); the National Institute of Allergy and Infectious Diseases (A167037 to ESR); from the National Institute of Dental and Craniofacial Research (DE17338 to ESR), and the Pennsylvania Department of Health (PA DOH Commonwealth Universal Research Enhancement Program) Tobacco Settlement grant to MF that "specifically disclaims responsibility for any analysis, interpretations or conclusions".

## Competing interests

The authors declare that they have no competing interests.

## Authors' contributions

KOS designed the study, conducted experiments, interpreted data and drafted the manuscript, MM conducted experiments, interpreted data and drafted the manuscript, MF performed microscopy and drafted the manuscript, APS recruited patients, collected samples and drafted manuscript, ON recruited patients, extracted patient tissues and drafted the manuscript, GB designed the study and drafted the manuscript, and ESR designed the study, interpreted data, drafted the manuscript, and supervised study. All authors have read and approved the final manuscript.
